# Breast mass as the initial presentation of esophageal carcinoma: a case report

**DOI:** 10.4076/1757-1626-2-7049

**Published:** 2009-07-07

**Authors:** Mohammad Tayefeh Norooz, Laleh Montaser-Kouhsari, Hamed Ahmadi, Mansour Jamali Zavarei, Parviz Daryaei

**Affiliations:** 1 Department of Oncology, Cancer Institute, Imam-Khomeini Hospital, Blouvar Keshavarz Street, Tehran University of Medical Sciences, Tehran, 1136746911, Iran; 2 Department of Pathology, Cancer Institute, Imam-Khomeini HospitalBlouvar Keshavarz Street, Tehran University of Medical Sciences, Tehran, 1136746911Iran

## Abstract

**Introduction:**

Esophageal cancer is considered as a fatal malignancy. It mostly metastasizes to lung, liver, and bone while breast metastasis has been rarely reported. This is the fifth report of metastatic breast cancer from esophageal cancer, which differs from previous reported cases in terms of initial presentation with metastatic breast mass and no metastatic involvement of other organs.

**Case presentation:**

We present a 35-year-old Caucasian woman who initially complained of a painful breast mass. Squamous pearls on cytologic evaluation suggested a metastatic lesion. Two months history of dysphagia was extracted through detailed interview with patient and further investigation revealed a stage IV esophageal squamous cell carcinoma.

**Conclusion:**

In this case, breast lesion as an unusual presentation of esophageal carcinoma emphasizes the great role of thorough medical history taking and cytologic study in evaluating an accidentally detected breast mass. The increasing reports of breast metastasis in patients with esophageal carcinoma necessitate the careful breast examination in visits after treatment of the primary tumor.

## Introduction

Esophageal carcinoma (EC) is considered as the sixth lethal malignancy [1]. The most common sites of metastatic involvement in EC are lung, liver and bone and it exceptionally metastasizes to the breast. Metastatic breast lesions are commonly detected in known cases of extramammary malignancies and usually manifests while there is a widespread metastasis to other organs [2]. There are only five reports of breast metastasis from esophageal squamous cell carcinoma (SCC) in literature so far. Interestingly, this is the first case of esophageal SCC, which presented with metastatic breast lesion as an initial manifestation and no other organ involvement.

## Case presentation

**Figure 1.**
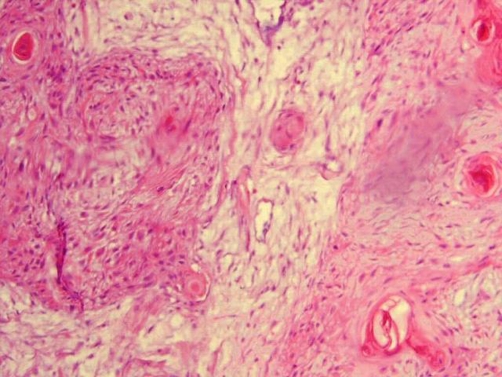
Breast histological sample (haematoxylin-eosin stain 40 x) shows the presence of squamous pearls.

**Figure 2.**
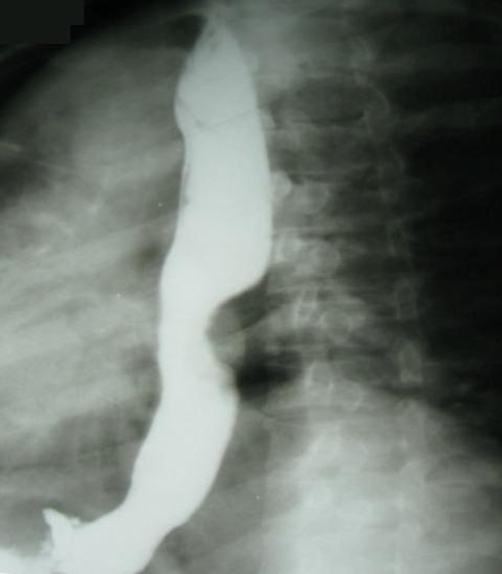
Barium swallow reveals the presence of a stenotic trait in the middle thoracic esophagus.

A 35-year-old Caucasian woman from northern Iran visited the Cancer Institute, Imam Khomeini Hospital in September 2008, with complaint of a painful lump in her right breast. She also mentioned an intermittent right parasternal pain, with spread to the back and a 6 kg weight loss. On examination, there was a 4 × 4.5 cm firm mass located just below the nipple, which was tender and mobile. Axillary and cervical chain lymph nodes were not palpable. Mammography revealed a single mass with vague margin in deep lower part of right breast, which had no microcalcification. Laboratory findings showed elevated Lactate Dehydrogenase (LDH) and Cancer Antigen (CA-125) plasma level, 364 U/ml (normal range 129-241 U/ml) and 186 ng/ml (normal range <35 ng/ml), respectively. The breast tumor was excised and histological evaluation revealed the presence of squamous pearls (Figure 1). Immunostaining was negative for estrogen and progesterone receptors and Human Epidermal Growth Factor Receptor-2 (HER-2). Thus, the diagnosis of metastatic squamous cell carcinoma was made and the patient was subjected to thorough evaluation to find out the primary site of the tumor. During the patient’s interview, we found out a 2-month history of dysphagia, primarily to solid foods with subsequent difficulty in liquid intake (grade IV), surprisingly not prominent to the patient. Her past medical and family history revealed no serious disease or malignancy. She did not mention any specific dietary habit or history of tobacco and alcohol use. In physical examination, she was not obese with body mass index of 21, and there was no palpable lymph node. Carcinoembryonic Antigen (CEA) was 18 μg/L (normal < 3 μg/L) and Carbohydrate Antigen 19-9 (CA19-9) was 180 U/ml (normal < 40 U/ml). All other laboratory parameters including CA-125 were within normal range. Barium swallow revealed a stricture with a marked irregularity in the middle part of esophagus lower to carina. A mass like lesion was also reported next to the stricture (Figure 2). Upper gastro-intestinal endoscopy showed a mass, 8 cm in diameter, placed 25 cm away from incisor teeth and a second smaller mass with fragile mucosa just under this area, which has narrowed the esophageal lumen. Biopsy specimens from both masses and esophageal wall revealed SCC. Chest computed tomography (CT) with contrast showed a stricture and wall thickening in distal part of the esophagus with dilation of proximal part. Lung parenchyma seemed clear. Abdomino-pelvic CT was normal with no sign of liver metastasis. With the diagnosis of esophageal SCC, transhiatal total esophagectomy was performed. One out of four extracted lymph nodes had tumor involvement. Histopathological examination confirmed the diagnosis of invasive malignancy (squamous cell type) in middle and lower parts of esophagus invaded into the adventitia. No vascular and perineural invasion was identified. The tumor was close to deep surgical margin (<40* objective field), with maximal thickness of 1.2 cm. Distal and proximal margins were free of tumor, with no evidence of tumor multicentricity. Therefore, the tumor was classified as pathologic Stage IV and T3N1M1, based on the TNM classification of malignant tumors. Thus, radiation with the total dose of 5000 cGy was introduced for the duration of 45 days. In addition, chemotherapy regimen consisted of 5-fluorouracil (1500 mg/m^2^ iv daily) plus cisplatin (45 mg/m^2^ iv daily) was administered at the first and last 3 days of radiation therapy.

## Discussion

Esophageal cancer is considered a fatal malignancy, which rapidly spreads throughout the body. Squamous cell carcinoma, as a major histological subtype, responsible for 70 percent of EC cases, predominantly involves the upper and mid-esophagus [3,4]. Early symptoms of EC such as transient sticking of foods, retrosternal discomfort or a burning sensation are nonspecific and subtle but dysphasia with subsequent weight loss, become apparent when the disease has been disseminated. In fact, 50-60% of EC tumors are unresectable at the time of diagnosis. Distant metastasis occurs in 26 percent of locally advanced ECs in the first 2 years of initial therapy, which mainly involves lung, skeletal system and liver. Similarly, in all four previous cases of EC. with breast invasion, the primary tumor diagnosis and treatment preceded the detection of metastatic lesion by a time interval between 2months and 2 years [3-5]. Notably, this is the first case of metastatic esophageal SCC in which the metastatic lesion as the presenting manifestation lead to identification of the primary tumor.

Metastatic breast masses account for only 0.4%-6.6% of all mammary malignancies. In fact, large amount of fibrous tissue with a moderately poor blood supply of the breast makes it an unusual site for metastatic lesions [6]. Metastatic breast masses could originate from any types of malignancies [7]. They dominantly arise from lymphoma, melanomas and rhabdomyosarcoma. Most of breast metastases are identified in known cases of extramammary malignancies [8,9]. Thus, the metastatic lesion of an unknown origin is by far the least possible diagnosis of an accidentally detected breast mass. However, breast metastasis could be the initial manifestation of an invasive tumor with mammary invasion in at least one quarter of occasions. In such situation, the metastasis may imitate primary benign or malignant neoplasms of the breast which could puzzle the physician and contribute in unnecessary surgical treatments [10].

Metastatic lesions however present some features that make them distinguishable from primary breast tumors. Most cases of mammary metastases were reported in younger age than primary breast cancer cases [6]. High blood flow of breast in young adults considered as the main culprit. Metastasis is usually a single, painless, mobile, ipsilateral and well-circumscribed tumor sited in the upper quadrants of the breast. On mammography, it runs the gamut from normal to different patterns of primary breast carcinoma. The most common mammographic characteristics are single or more well-defined masses with high density with regular margin and no evidence of microcalcification or skin thickening. Cytologic features of the breast metastasis may vary from unusual types such as small cell carcinoma, melanoma, lymphoma, or squamous and large cell carcinoma to those unrecognizable from primary adenocarcinoma of the breast [7].

Painfulness of the mass, atypical location and irregular margins on mammography made the differentiation with primary breast neoplasm even more difficult in this case and if it wasn’t for the squamous pearls on pathology as well as a thorough medical history, the patient would be misdiagnosed as having the primary breast cancer.

Patients with metastatic EC have a median survival time of six months and only 14% of patients will survive after 5 years because of the local or distant recurrence. Chemotherapeutic treatment provides an overall survival of 12 months [4,7]. Even after complete resection of esophageal tumor, as the main treatment modality, the survival rate increases up to 35 percent, on average. Prognosis for the breast metastasis is very poor and most of the patients survive less than one year [7]. Surgical resection of breast metastasis suffices if metastases in other sites are not present or being under controlled [4]. Therefore, after excising the breast tumor, we only applied treatment regimen for primary tumor. To date, our patient has acceptable health condition 6 months after the treatment with no complication and no sign of recurrence.

## Conclusion

This is the first case of advanced esophageal SCC, which presented with metastatic breast lesion, no other organic involvement and atypical clinical and radiographic features of the metastatic lesion. Detailed medical history taking and cytologic study are of at most importance in evaluating an accidentally detected breast mass in order to apply proper treatment. The increasing reports of breast metastasis in EC patients necessitate the careful breast examination in this group of patients.

## Abbreviations

CA19-9: Carbohydrate Antigen 19-9
CA-125: Cancer antigen-125
CEA: Carcinoembryonic antigen
EC: Esophageal carcinoma
HER-2: Human epidermal growth factor receptor-2
LDH: Lactate dehydrogenase
SCC: Esophageal squamous cell carcinoma

## References

[bib-001] Iwanski G, Block A, Keller G, Muench J, Claus S, Fiedler W, Bokemeyer C (2008). Esophageal squamous cell carcinoma presenting with extensive skin lesions: a case report. J Med Case Reports.

[bib-002] Ribeiro-Silva A, Mendes CF, Costa IS, de Moura HB, Tiezzi DG, Andrade JM (2006). Metastases to the breast from extramammary malignancies: a clinicopathologic study of 12 cases. Pol J Pathol.

[bib-003] Miyoshi K, Fuchimoto S, Ohsaki T, Sakata T, Takeda II, Takahashi K, Ohkawa T, Murata T, Kuwada Y (1999). A Case of Esophageal Squamous Cell Carcinoma Metastatic to the Breast. Breast Cancer.

[bib-004] Santeufemia DA, Piredda G, Fadda GM, Cossu Rocca P, Costantino S, Sanna G, Sarobba MG, Pinna MA, Putzu C, Farris A (2006). Successful outcome after combined chemotherapeutic and surgical management in a case of esophageal cancer with breast and brain relapse. World J Gastroenterol.

[bib-005] Nielsen M, Andersen JA, Henriksen FW, Kristensen PB, Lorentzen M, Ravn V, Schiodt T, Thorborg JV, Ornvold K (1981). Metastases to the breast from extramammary carcinomas. Acta Pathol Microbiol Scand [A].

[bib-006] Silverman JF, Feldman PS, Covell JL, Frable WJ (1987). Fine needle aspiration cytology of neoplasms metastatic to the breast. Acta Cytol.

[bib-007] Shiraishi M, Itoh T, Furuyama K, Yamasaki S, Shimada Y, Hosotani R, Nakashima Y, Imamura M (2001). Case of metastatic breast cancer from esophageal cancer. Dis Esophagus.

[bib-008] Komorowski AL, Wysocki WM, Mitus J (2005). Metastasis to the breast-a clinical challenge in outpatient. Acta Chir Belg.

[bib-009] Bartella L, Kaye J, Perry NM, Malhotra A, Evans D, Ryan D, Wells C, Vinnicombe SJ (2003). Metastases to the breast revisited: radiological-histopathological correlation. Clin Radiol.

[bib-010] Gupta D, Merino MI, Farhood A, Middleton LP (2001). Metastases to breast simulating ductal carcinoma in situ: report of two cases and review of the literature. Ann Diagn Pathol.

